# Toxicological investigation of lilial

**DOI:** 10.1038/s41598-023-45598-y

**Published:** 2023-10-28

**Authors:** Eva Jablonská, Zdeněk Míchal, Bára Křížkovská, Ondřej Strnad, Van Nguyen Tran, Tereza Žalmanová, Jaroslav Petr, Jan Lipov, Jitka Viktorová

**Affiliations:** 1 Department of Biochemistry and Microbiology, University of Chemistry and Technology, Prague, Technická 5, 166 28 Prague 6, Czech Republic; 2 Department of Biology of Reproduction, Institute of Animal Science, Prague 10-Uhrineves, Czech Republic

**Keywords:** Biochemistry, Molecular biology

## Abstract

Lilial (also called lysmeral) is a fragrance ingredient presented in many everyday cosmetics and household products. The concentrations of lilial in the final products is rather low. Its maximum concentration in cosmetics was limited and recently, its use in cosmetics products was prohibited in the EU due to the classification as reproductive toxicant. Additionally, according to the European Chemicals Agency, it was under assessment as one of the potential endocrine disruptors, i.e. a substance that may alter the function of the endocrine system and, as a result, cause health problems. Its ability to act as an androgen receptor agonist and the estrogenic and androgenic activity of its metabolites, to the best of our knowledge, have not yet been tested. The aim of this work was to determine the intestinal absorption, cytotoxicity, nephrotoxicity, mutagenicity, activation of cellular stress-related signal pathways and, most importantly, to test the ability to disrupt the endocrine system of lilial and its Phase I metabolites. This was tested using set of in vitro assays including resazurin assay, the CHO/HPRT mutation assay, γH2AX biomarker-based genotoxicity assay, qPCR and in vitro reporter assays based on luminescence of luciferase for estrogen, androgen, NF-κB and NRF2 signalling pathway. It was determined that neither lilial nor its metabolites have a negative effect on cell viability in the concentration range from 1 nM to 100 µM. Using human cell lines HeLa9903 and MDA-kb2, it was verified that this substance did not have agonistic activity towards estrogen or androgen receptor, respectively. Lilial metabolites, generated by incubation with the rat liver S9 fraction, did not show the ability to bind to estrogen or androgen receptors. Neither lilial nor its metabolites showed a nephrotoxic effect on human renal tubular cells (RPTEC/TERT1 line) and at the same time they were unable to activate the NF-κB and NRF2 signalling pathway at a concentration of 50 µM (HEK 293/pGL4.32 or pGL4.37). Neither lilial nor its metabolites showed mutagenic activity in the *HPRT* gene mutation test in CHO-K1 cells, nor were they able to cause double-strand breaks in DNA (γH2AX biomarker) in CHO-K1 and HeLa cells. In our study, no negative effects of lilial or its in vitro metabolites were observed up to 100 µM using different in vitro tests.

## Introduction

**Figure 1:**
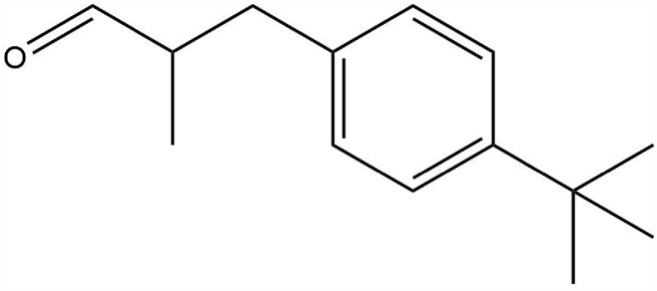
3-(4-(tert-butyl)phenyl)-2-methylpropanal (ChemDraw 16.0).

Almost all of us encounter perfumes, cosmetics, or cleaning products every day. A synthetic fragrance ingredient frequently used in such preparations is 3-(4-(tert-butyl)phenyl)-2-methylpropanal (Fig. 1), abbreviated p-BMHCA, also known as lysmeral, lilial, or lily aldehyde (CAS No 80-54-6). In the manufacturing process, the substance is obtained as racemic mixture (1:1) of the two enantiomers. This substance is classified as a strong allergen (skin sensitizer)and reproductive toxicant (CMR1B classification). The evaluation as endocrine disrupting chemical by ECHA was inconclusive^1^.

According to Usta et al.^2^, lilial induced a toxic effect on mitochondria that caused a decrease in the viability of HaCaT cells. It was claimed to target respiratory chain complexes, inhibit complexes I and II of the electron transport chain, increase the generation of reactive oxygen species, and decrease the level of intracellular ATP^2^. The repeated dose toxicity data on several species after ingestion was investigated. Oral administration at doses of ≥ 50 mg/kg/day (rats) and ≥ 200 mg/kg/day (dogs) reduced body weight, had signs of clinical toxicity, and induced systemic toxicity such as decreasing in plasma cholinesterase activity levels^3^. However, no statistical significance and histopathological correlations were observed.

Lilial did not show genotoxicity and mutagenicity in a variety of further mentioned in vitro studies. No genotoxic and mutagenic potential was found in bacteria including *S. typhimurium* and *E. coli,* and in CHO and Chinese hamster V79 cells^4^. Similarly, lilial did not induce genotoxicity at the chromosomal level (clastogenicity or aneuploidy) or single-strand breaks at concentrations up to 500 μM^5^. However, in another study “in ovo” using fertilized eggs of white turkey (*Meleagris gallopavo*), lilial was reported to induce significant DNA strand breaks in the comet assay (2.0-fold) at 10 mg/egg, but this occurrence was possibly caused by an indirect mode of action (mediated by oxidative stress) of the chemical instead of chemical-mediated DNA damage^6^.

An endocrine disruptor is an exogenous substance or mixture that alters the function of the endocrine system and consequently causes adverse health effects in an intact organism, its progeny, or subpopulations^7^. Regarding endocrine disrupting effects, lilial was under assessment as endocrine disrupting^1^. Lilial exhibited estrogenic activity in MCF-7 human breast cancer cells in vitro^8^. Nevertheless, the effect of lilial was very weak compared to estrogen (only 2.4 doublings with 100 µM vs. 5.2 doublings with 10 nM estrogen compared to 1.8 doublings of unaffected control). However, there were no adverse effects on fertility and reproductive performance in the parental rats F0 and F1 in vivo^3^. Furthermore, the mode of action of lilial in sensitive species appears to be not dependent on an endocrine effect but related to overt toxicity to seminiferous tissues^4^.

Skin and eye irritation properties are also assigned to this compound. Pure lilial causes eye and skin irritation in rabbits^4^. The dermal absorption values were 13.5% for hydroalcoholic-based fragrances and deodorant/antiperspirant products, 8.9% for oil-in-water-based products including make-up products, body lotions, hair styling and bath cleaning products, and 10.5% for water-in-oil-based products such as face and hand cream products^3^. Lalko et al.^9^ demonstrated the sensitization potential of lilial using the Local Lymph Node Assay. Lilial would be expected to react with skin proteins^10^. In a human maximization test, no skin sensitization reactions were observed at a concentration of 4%. However, in another test, lilial showed skin sensitization reactions at a concentration of 5%, but this may be caused by the spread of phenylacetaldehyde^3^. Lilial is listed as one of 26 fragrance ingredients that cause allergic contact dermatitis, which must be declared on cosmetic products^11^. Allergic contact dermatitis is a form of contact dermatitis caused by contact with chemical sensitizers^12^. Many signalling pathways are involved in allergic contact dermatitis including mitogen-activated protein kinases (MAPK), nuclear factor kappa B (NF-κB), and nuclear factor erythroid-2-related factor 2 (NRF2)^13^. NF-κB competes with NRF2 for the binding of CREB-binding proteins in the nucleus leading to the inactivation of the NRF2 pathway. Moreover, NF-κB impedes the NRF2 signalling by recruiting histone deacetylases. In contrast, heme oxygenase 1, a downstream gene of NRF2, can inhibit the nuclear translocation of NF-κB pathway^14^. Generally, the interplay between the NRF2 and NF-κB pathways plays an important role in the regulation of oxidative stress and inflammation. In the study by Ade et al.^15^, a significant increase in the level of NRF2 protein in THP-1 cells treated with lilial (650 µM) for 5 h suggested that the NRF2 pathways are activated by lilial. In 2022, OECD released a test guideline 442D for in vitro skin sensitisation^16^, which describes two in vitro ARE-Nrf2 luciferase test methods (KeratinoSens and LuSens test method), a reporter cell-based approach based on the regulatory pathway involving Nrf2^17^. Lilial was negative in the KeratinoSens assay^18^ Even the metabolites of lilial (*i.e.* lilial acid, hydroxylated lilial acid and p-tert-butylbenzoic acid) are expected to have a lower skin sensitizing potential than lilial itself, which is an aldehyde, the metabolites have not been studied in this manner, in other words, there are no publications on the lilial metabolites-activated NRF2 and NF-κB pathways.

Use of lilial was first restricted in European Union based on the skin sensitisation properties. The presence of lilial had to be indicated on the products if its concentration in products that remain on the skin (such as creams, lotions, and deodorants) exceeds 0.001% or 0.01% in products that were washed off after use (soaps, shampoos). Additionally, its total concentrations in products must not exceed given limits—for example, 0.05% in body creams, 0.1% in bath preparations, or 1.42% in perfumes^4^. The maximum allowed concentration of 1.42% in perfumes corresponded to a molar concentration of 65 mM (assuming that 1.42% is a percentage by volume). In 2022, the use of lilial in cosmetic products was prohibited based on the CMR 1B classification in the EU^19^ Uni. However, there are a number of publications contradicting each other and presenting inconsistent research data.

In this context, the present study aims to evaluate the intestinal absorption, acute toxicity, nephrotoxicity, mutagenicity, the ability of endocrine disruption, and activation of lilial cellular stress-related signal pathways. Potential genotoxicity, mutagenicity, nephrotoxicity, and NF-κB and NRF2-ARE signal pathway were assessed using a CHO/HPRT mutation assay, γH2AX biomarker-based genotoxicity assay, and in vitro reporter assays. The estrogenic and androgenic activity was determined by the binding ability of the lilial to receptors compared to 17β estradiol (E2) and dihydrotestosterone (DHT) in Hela9903 cells and MDA-kb2 cells. In addition, for all tests, the lilial metabolites produced from incubation with the rat liver S9 fraction were also included.

## Material and methods

### Material

The following standards and chemicals were purchased from Merck (Saint Louis, Missouri, USA): Dulbecco’s modified Eagle’s medium—high glucose (DMEM, D0819), minimum essential medium (MEM, M0446), dimethylsulfoxide (DMSO), stable l-glutamine solution (G8541), l-proline, fetal bovine serum (FBS), trypsin-2,2′,2″,2‴-(ethane-1,2-diyldinitrilo)tetraacetic acid (EDTA) solution ,resazurin sodium salt, dextran treated charcoal fetal bovine serum (DCC-FBS, F6765), 17β estradiol (E2), dihydrotestosterone (DHT), ethylmethanesulfonate (EMS), 3-methylcholanthren (3-MC), 6-thioguanine (6-TG), non-essential amino acid solution (100× , NEAA), tert-butyl hydroquinone, tumor necrosis factor-α human (TNFα), lilial (43884, contains ~ 0.01% stabilizer (Pyridine/MEHQ: 1/20)).

The following standards and chemicals were purchased from Thermo Fisher Scientific (Waltham, Massachusetts, USA): minimum Essential Medium without phenol red (MEM, 51200046), Roswell Park Memorial Institute (RPMI) 1640 medium without phenol red, geneticin (G418), blasticidin S, hypoxanthine-aminopterin-thymidine (HAT) medium.

ProxUp2 medium was purchased from Evercyte (Wien, Austria), S9 extract of rat liver activated with Aroclor (40 mg of proteins/ml, S9) from Trinova Biochem (Gießen, Germany) and ONE-Glo EX luciferase assay system from Promega (Madison, Wisconsin, USA).

Lilial solutions were prepared in DMSO. 100 mM stock solution was prepared by mixing 10,6 µl of lilial (10 mg) with DMSO up to final volume of 489 ml.

### Cell lines

Caco-2 (human colorectal adenocarcinoma, ATCC, HTB-37) for intestinal absorption were cultivated in EMEM supplemented with 10% FBS and 1% antibiotic mixture (penicillin, 100 IU/ml and streptomycin, 100 g/ml) and incubated at standard conditions (37 °C, 5% CO_2_, humidified atmosphere).

CHO-K1 (Chinese hamster ovarian cells, ECACC, 85051005) used for the cytotoxicity assay and for the mutation assay were cultivated at standard conditions (37 °C, 5% CO_2_, humidified atmosphere) in DMEM supplemented with 10% FBS + l-Pro (final concentration 35 mg/l). This medium was chosen because it does not contain hypoxanthine and, therefore, can be also used during the selection. The concentration of l-proline (essential for CHO-K1 cells) was identical as in Ham´s medium.

HeLa (human cervical adenocarcinoma, ATCC, CCL-2) used for the genotoxicity assay were cultivated at standard conditions (37 °C, 5% CO_2_, humidified atmosphere) in EMEM (MEM + NEAA) supplemented with 2 mM stable glutamine and 10% FBS.

HeLa9903 (stably transfected subclone of HeLa cells, ECACC, 11,033,105) and MDA-kb2 (human mammary gland adenocarcinoma, ATCC, CRL-2713) used for the cytotoxicity assay and for the transactivation assays were incubated at standard conditions (37 °C, 5% CO_2_, humidified atmosphere) in MEM and RPMI, respectively; both without phenol red and supplemented with 2 mM stable glutamine and 10% DCC-FBS. HeLa9903 cells were regularly treated with a combination of geneticin (800 µg/ml) and blasticidin S (16 µg/ml) and MDA-kb2 cells were regularly treated with geneticin (500 µg/ml) to maintain their responsivity.

RPTEC/TERT1 (hTERT-immortalized human proximal tubular epithelial cells, Evercyte, CHT-003-0002) used for the nephrotoxicity assay were cultivated at standard conditions (37 °C, 5% CO_2_, humidified atmosphere) in ProxUp2 medium.

HEK293 (human embryonic kidney cells, ATCC, CRL-157) used for transfection and cellular signalling assays were cultivated at standard conditions (37 °C, 5% CO_2_, humidified atmosphere) in DMEM supplemented with 2 mM stable glutamine and 10% FBS.

Cells were regularly passaged when reached 80% confluence using standardized trypsin/EDTA detaching method. All the experiments were performed with the cells between passage 5 and 20 except for Caco-2 cells, where passages 30–50 was used. All cells were regularly controlled for the presence of mycoplasma (MycoAlert Mycoplasma Detection Kit, Lonza, Czech Republic) and were regularly authenticated using short tandem repeat (STR) profiling.

### Intestinal absorption

Intestinal absorption of lilial was determined according to previously published work^20^ using intestinal layer prepared from differentiated Caco-2 cells in vitro. Lilial was applied in 1 mM concentration and after 4 h, its amount in apical and basolateral medium and also in Caco-2 cells was determined by GC/HRMS using Agilent system 7500 GC Q-TOF + 7890 with following parameters: inlet 150 °C, splitless; carrier gas He, 1.4 ml/min; GC column DB-1 5 m × 0.32 mm + 1.5 m 0.15 mm restrictor; oven 40 °C–5 min–20 °C/min–290 °C; MS EI + 70 eV; source 230 °C; scan 30–500 a.m.u., 5 scans/s; injection volume 1 ul, Agilent 7693 AS. The apparent permeability coefficient (Papp) was calculated from the permeation rate and compound concentration as follows:

$$P_{app} = \frac{{{\text{dQ}}/{\text{dt}}}}{{{\text{A*}}c_{0} }}$$

where dQ represents concentration change of lilial present in the basolateral medium (nM), dt is duration of the experiment (s), A is area of transwell (1.13 cm^2^), c_o_ is initial concentration of lilial (nM).

### Metabolic activation of lilial

S9 mix for metabolic activation was composed of: 50 mM NaH_2_PO_4_·2H_2_O; 50 mM Na_2_HPO_4_·2H_2_O; 4 mM·NADP; 5 mM glucose-6-phosphate; 33 mM KCl; 8 mM·MgCl_2_·6H_2_O; 10 mM CaCl_2_·2H_2_O; 9% (v/v) S9 according to^21–23^. For genotoxicity testing (4 h incubation), the S9 mix was added to the medium in the ratio 1:5 and lilial was activated directly in the petri dish/well with the cells. For other test (24 h incubation), metabolic activation was performed separately according to^24^ and metabolites were added to the cells dissolved in DMSO. Briefly, 100 µl of S9 mix was mixed with 1 µl of lilial (1 mM) in a microtube and incubated at 37 °C with shaking for 1 h. Thereafter, 50 µl of ACN was added to the solution to stop the reaction. The solutions were placed for 15 min on ice and subsequently centrifuged (5 min, 18,000×*g*, 4 °C) to remove precipitated proteins. The supernatant was transferred to new microtubes and completely evaporated overnight at 37 °C using vacuum evaporator (Labconco, USA). The obtained extracts were further dissolved in 10 µl of DMSO. The resulting solution was used for assessment of estrogenic activity, nephrotoxicity and NF-κB and NRF2 signal pathway activation. As an inactivated control, mixture of S9 mix and lilial with 50 µl of ACN added to the reaction immediately to prevent the reaction was also prepared. At the end of the procedure (dissolving in 10 µl of DMSO), the concentration of lilial in this solution should be 10 mM.

Metabolic activation was verified using GC/HRMS as described above.

### Cytotoxicity

One day before the exposition, cells (CHO-K1 and HeLa9903 cells) were trypsinized and resuspended in an appropriate cultivation medium to create a suspension with a concentration of 1 × 10^5^ cells per ml. Thereafter, 100 µl of the cell suspension was seeded into a 96-well plate. After one day, the medium was replaced by the tested solutions. Six replicates (wells) were used for each sample. Sole cultivation medium with 5% FBS served as a control (unaffected cells). Cultivation medium with DMSO (0.1% and 1%) served as a vehicle control. 0.25% Tween-20 served as a positive (toxic) control.

After one day of incubation with the tested solutions, cell viability was evaluated using resazurin assay^25^. Extracts were removed and resazurin solution (final concentration 25 µg/ml) in the cultivation medium with 10% FBS without phenol red was added. After incubation (1–4 h, depending on the type of experiment), fluorescence at 560/590 nm (excitation/emission) was measured. Cytotoxicity of the extracts was depicted as a percentage of viability of the control.

### Estrogenic and androgenic activity

The test for estrogenic activity with HeLa9903 was performed in accordance with EPA^26^ and OECD standard^27^. Briefly, cells were seeded in white 96-well plates (Corning, New York, United States) in the density of 1 × 10^5^ cells per well. 3 h after the seeding, solutions of lilial (1–100 µM) and E2 (positive control, 0.1 pM–10 nM) were added to the cells. The concentration of DMSO in all solutions was 0.1%. Sole medium served as a negative control (NC) and 0.1% DMSO in medium served as a vehicle control (VC).

After 24 h incubation, ONE-Glo EX Luciferase Assay System solution was added to the wells and luminiscence was measured using SynergyH1 (BioTek, Winooski, Vermont, USA).

The test for androgenic activity with MDA-kb2 was performed in a similar manner as described above with following differences: The seeding density was 2.5 × 10^4^ cells per well and dihydrotestosterone (0.01 nM–100 µM) was used as a positive control.

Results were expressed as fold induction (FI), i.e. fold increase compared to vehicle control. According to EPA^28^, FI of positive control should be above 4 to meet the performance standard.

### Mammalian cell gene mutation assays (CHO/HPRT mutation assay)

The test with metabolic activation was performed as described in the OECD protocol^29^, the ASTM standard^23^ and other protocols^30,31^ and publications^32^. For the test, cryopreserved CHO-K1 cells after the mutant cleansing were used. Mutant cleansing was performed according to^30^, i.e*.* cells were cultivated three days in HAT medium, one day in HT medium and then frozen.

One passage after the thawing, 1 × 10^6^ cells were seeded in 60 mm cultivation plates as described in^32^. After 24 h, the medium was removed, plates were washed with PBS to remove residual FBS and lilial dissolved in DMSO was added to the medium without FBS. The final concentration of lilial was 50 µM (the highest non-toxic concentration) and 500 µM (the non-toxic concentration of metabolically activated lilial). The final concentration of the solvent, DMSO, was 1%. The S9 mix (composition described in chapter 2.4) was added to the medium in the ratio of 1:5. EMS at final concentration 2 mM as recommended in^31^ served as a positive control. 3-MC at final concentration 7.5 mM^22^ served as a positive control for metabolic activation. 1% DMSO was used as a VC. Sole cultivation medium served as a negative control (NC). After 4 h, the medium with tested substances was removed, plates were washed with PBS and fresh medium with 10% FBS was added.

During the expression period (1 week), the cells on all 60 mm plates were passaged once. On day 8, cells were trypsinized, resuspended, counted and 2 × 10^5^ cells of each plate were seeded on 60 mm plates in triplicates in the presence of a selective agents, 6-TG (30 µM) as described in^32^. The concentration of 6-TG was higher than recommended by ASTM, because we did not use dialysed serum and purines from serum could interfere with 6-TG. 300 cells without the selective agent were seeded in a parallel to evaluate plating efficiency (PE). On day 15, the plates were washed with PBS, cells were fixed with ice cold methanol for 10 min and stained with 0.05% crystal violet in water for 30 min^33^. After washing with water, formed colonies were counted using Schuett Count (Schuett-biotec, Göttingen, Germany). Calculations were done as described in^29^. PE was calculated according to a general Eq. 1. Particular PE at selective conditions was calculated according to Eq. 2. Particular PE at non-selective conditions was calculated according to Eq. 3. Mutation frequency (MF) was calculated according to Eq. 4.

1 $$PE = \frac{number\, of\, colonies\, formed}{{number\, of\, cells\, plated}}$$

2 $$PE_{selective} = \frac{{number\, of\, mutant\, colonies \left( {NMC} \right)}}{200,000}$$

3 $$PE_{non - selective} = \frac{number\, of\, colonies\, formed}{{300}}$$

4 $$MF = \frac{{PE_{selective\, conditions} }}{{PE_{non - selective\, conditions} }}$$

### γH2AX biomarker-based genotoxicity assay

Genotoxicity of lilial and its metabolites was evaluated using HCS DNA Damage Kit (Invitrogen, Waltham, Massachusetts, USA). Briefly, HeLa and CHO-K1 cells were seeded at a concentration of 1 × 10^5^ cells/ml into the 96-well plates. CHO-K1 cells were used for comparison with HPRT test. HeLa cells were recommended by the manufacturer of the kit. After 24 h, cells were rinsed with PBS and cultivation medium (“Cell lines” Section) without ATB, with a reduced content of FBS (5%) was added. 30 µM valinomycin (in DMSO) was used as a positive control Samples were applied in the presence of non-toxic sample concentrations, i.e. 100 µM either with or without S9 mix. After 4 h or 24 h incubation, the cells were fixed and stained according to HCS DNA Damage Kit instruction using Hoechst 33342 (350/461 nm binds to DNA; cell count), Alexa Fluor^®^ 555 (555/565 nm secondary antibody; binds to primary antibody), an antibody against phosphorylated H2AX (Ser139, DNA damage, which is induced in response to double-strand break formation). Cells were photographed using a fluorescence microscope (Olympus IX83); filters: DAPI (500 ms) and Cy3 (500 ms). Bioimage analysis in ImageJ software was performed as described previously^34^.

### Nephrotoxicity

RPTEC/TERT1 cells were seeded at a concentration of 2 × 10^5^ cells/ml in ProxUp2 medium into 60 mm Petri dishes. After 24 h, the cells were washed with PBS and lilial and its metabolites (100 µM) were applied in fresh ProxUp2 medium. The cells were incubated with samples for 48 h, after which the cells were washed with PBS and dishes were frozen in − 160 °C. The RNA was isolated using RNeasy Plus Mini Kit (Qiagen, Hilden, Germany). The RNA concentration and purity was determined with NanoDrop One (ThermoFisher Scientific) spectrophotometr. The cDNA synthesis was performed from 0.1 µg of total RNA with an iScript Reverse Transcription Supermix (Bio-Rad, Hercules, California, USA). Quantitative real-time PCR (qPCR) was performed using the qPCR Bio-Rad C100 Touch System (Bio-Rad) with a 96-well block. The reaction mixture consisted of 1 µl of specific 20 × PrimePCR Assay (Bio-Rad), 10 µl of 2 × SsoAdvance Universal Supermix (Bio-Rad), 2 µl of cDNA sample, 7 µl of nuclease-free water. The final reaction volume was 20 µl.

PrimePCR Assays used: actin, beta (ACTB, qHsaCED0036269); glyceraldehyde-3-phosphate dehydrogenase (GAPDH, qHsaCED0038674); metallopeptidase inhibitor 1 (TIMP1, qHsaCID0007434); importin 8 (IPO8, qHsaCED0005354); interleukin 6 (IL6, qHsaCED0044677); cystatin C (CST3, qHsaCID0012666); interleukin 8 (IL8, qHsaCED0046633); clusterin (CLU, qHsaCID0012475); heme oxygenase (decycling) 1 (HMOX1, qHsaCID0022141).

Cycling parameters were: initial denaturation at 95 °C for 2 min, followed by 40 cycles of 95 °C for 5 s and 60 °C for 30 s. Fluorescence values were acquired after each extension phase. Samples were analyzed in triplicate, and samples with a standard deviation of duplicates > 0.2 Ct were re-analyzed. A non-template control containing nuclease-free water instead of cDNA as well as non-RT control containing purified RNA without reverse transcription was used. The real-time PCR study followed the MIQE guidelines^35^. Relative transcript levels of the estimated genes in the cell lines were compared using the software CFX Maestro Software 2.3 (Bio-Rad).

### NF-κB and NRF2-ARE signal pathway activation

Activation of signal pathways by lilial and its metabolites was evaluated using reporter assays. HEK293 cells were transiently transfected with reporter plasmid pGL4.32 (luc2P/NF-κB-RE/Hygro, Promega Corporation, Madison, Wisconsin, USA) or pGL4.37 (luc2P/NRF2-ARE/Hygro) using FuGENE (Promega Corporation). Plasmid pEGFP-C1 carrying the gene for green fluorescent protein (GFP) was co-transfected with the reporter plasmids as a normalization plasmid enabling normalization of the signal to transfection level. To test the activation of NF-κB pathway, TNFα (in the range from 13 to 200 pg/ml) as a positive control or samples (50 µM) were incubated with the cells for 5 h. To test the activation of NRF2-ARE pathway, tert-butyl hydroquinone (TBHQ, in the range from 4 to 63 µM) as a positive control or samples (50 µM) were incubated with the cells for 18 h. The fluorescence (488/513 nm) was determined using SpectraMax i3x Multi-Mode Microplate Reader (Molecular Devices, San Jose, California, USA). Subsequent quantification of luciferase activity was performed using the Bright-Glo™ Luciferase Assay System (Promega Corporation) according to the manufacturer’s instructions.

## Results and discussion

The present study evaluates the intestinal absorption, acute toxicity, nephrotoxicity, mutagenicity, the ability of endocrine disruption, and activation of lilial cellular stress-related signal pathways. For clarity, the methods used and the results are shown in Table 1.

**Table 1:** Summary of the in vitro cell-based methods used and results obtained.

Assay	Technique	Cell line	Concentration tested	Results
Intestinal absorption	intestinal barrier model in vitro	Caco-2	1 mM	Recovery = 79.5% P_app_ = 21 × 10^–6^ cm/s
Cytotoxicity	Cell viability (resazurin assay)	HeLa9903	1 nm–100 µM	Non toxic up to 100 µM
		CHO-K1	5 nm–500 µM	≥ 250 µM toxic Metabolites non toxic up to 500 µM
Estrogenic activity	Transactivation luciferase assay (stably transfected cell lines)	HeLa9903	1 nm–100 µM	Non active up to 100 µM
Androgenic activity		MDA-kb2	1 nm–100 µM	Non active up to 100 µM
Mutation assay	CHO/HPRT mutation assay	CHO-K1	50 µM	Non mutagenic
γH2AX biomarker-based genotoxicity assay	Immunofluorescence microscopy	HeLa	100 µM	Non genotoxic
		CHO-K1	100 µM	Inconclusive
Nephrotoxicity	RT-qPCR	RPTEC/TERT1	100 µM	Non nephrotoxic
NF-κB signal pathway activation	Transactivation luciferase assay (transiently transfected cell lines)	HEK293	100 µM	Non activator
NRF2-ARE signal pathway activation			100 µM	Non activator

### Intestinal absorption

The determination of gastrointestinal permeability is a key property in predicting the intestinal absorption and toxicity of many substances, keeping in mind that oral uptake is not of such relevance for lilial as a cosmetic ingredient. Potentially dangerous substances that cannot cross the intestinal layer do not pose a significant risk compared to substances that cross well. The use of in vitro models finds application, especially in efforts to reduce animal testing according to 3R principles. For this reason, the use of an intestinal barrier model created in vitro from differentiated Caco-2 cells (epithelial cells isolated from colon tissue) in transwells with defined pores and properties is becoming increasingly popular. Here, we determined the permeability of the lilial (Table 2) through Caco-2 cells. Recovery of lilial determined in all compartments was almost 80% indicating good solubility and low metabolism by the Caco-2 cells. The apparent permeability coefficient (P_app_) was calculated as 21 × 10^−6^ cm/s, suggesting high absorption in vivo^36^. In vitro dermal absorption studies of lilial have been performed in rat (78.4%) and guinea pigs (25.7%) at 1% p-BMHCA in ethanol applied at 120 µg substance/cm^2^ onto 5 cm^2^ skin for 16 h. In vivo dermal absorption of lilial was tested on 3 human volunteers for 6 h. A mean of 1.4% of applied dose was detected in urine within 24 h (below detection limit in urine samples of later time points and in all faeces and blood plasma samples) and high topical application recovery of lilial was also observed in the range of 71 ± 10%^4^.

**Table 2:** Detected amounts of lilial in different compartments of the Caco-2 cell system mimicking the intestinal barrier.

Fraction	Lilial (%)
Apical medium	32.3 ± 2.0
Caco-2 cells	13.1 ± 5.2
Basolateral medium	34.1 ± 3.4
Recovery	79.5%

The major uptake route of lilial is dermal and potentially via inhalation for some consumer products. Thus, ingestion involving intestinal uptake is not of such relevance for the toxicity of lilial for humans.

### Metabolic activation

The potential toxicity and adverse reactions do not result only from acute toxicity of the compound, but very often, the original non-toxic substances are transformed into toxic products by subsequent metabolic activation. Metabolic activation is a key step for the toxicity of most genotoxic carcinogens^37^. Metabolic activation is usually mediated by hepatic cytochrome P450, and leads to the generation of reactive or toxic metabolites that bind to cellular macromolecules such as glutathione, proteins, or DNA^38^ or could interact with endocrine or signalling pathways. Unfortunately, the testing of metabolically activated substances is still not a routine part of the toxicological safety assessment of substances.

**Figure 2:**
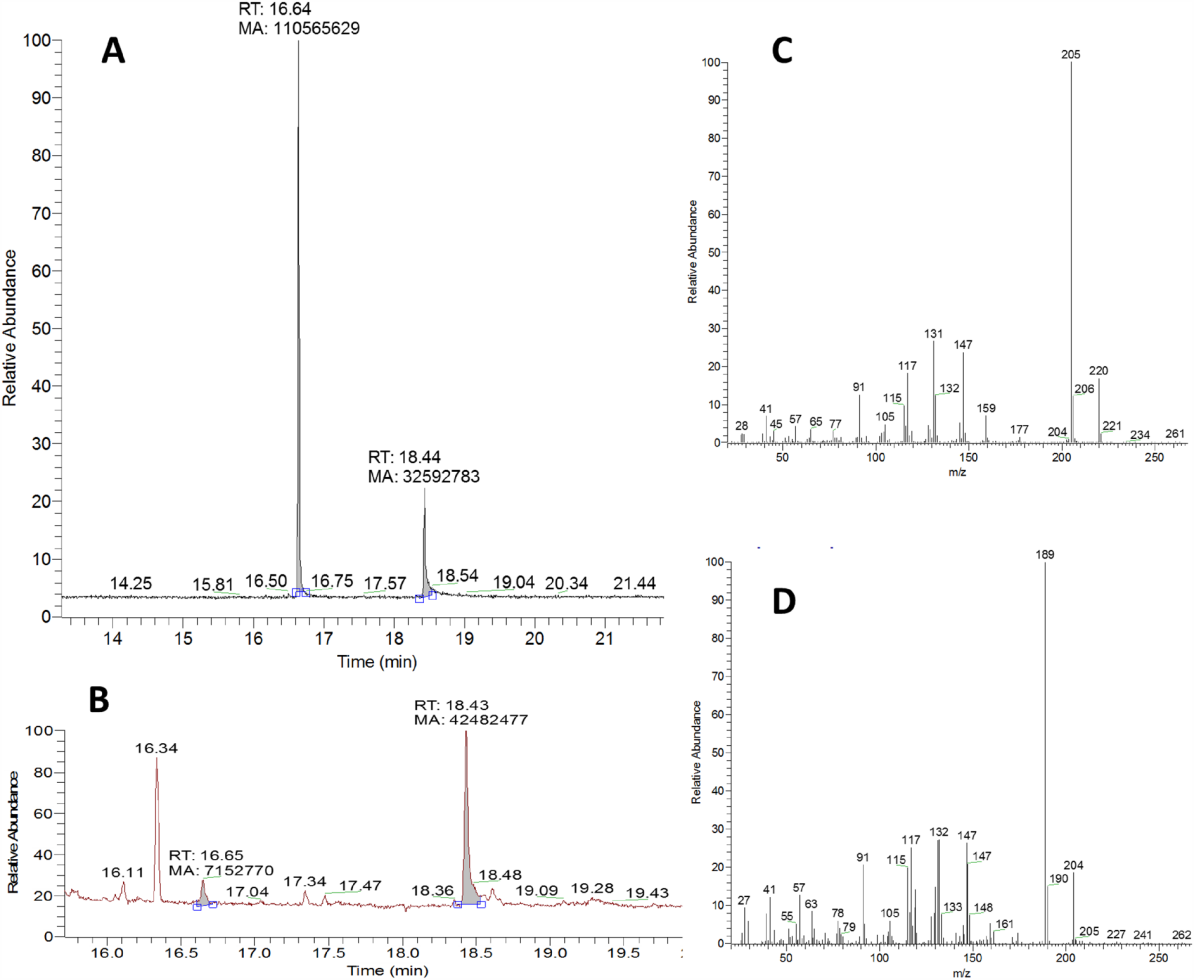
Retention and mass spectra of GC/MS analysis of lilial (**A**) and its metabolites (**B**). (**C**) spectrum of lilial, (**D**) spectrum of lysmerylic acid.

In order to verify the metabolic activation, we firstly determined the retention time of the lilial itself. Figure 2A shows two peaks with retention times of 16.64 and 18.44 min. According to the mass spectrum of the first peak (Fig. 2C), this substance was identified as lilial, the mass spectrum of the second peak (Fig. 2D) then corresponded to its metabolite–lysmerylic acid. The lilial batch used for the studies (retention time (RT) 16.64 min) contained already around 20% of lysmerylic acid (RT 18.44 min; Fig. 2) which is either an impurity of lilial and/or an oxidation product during storage. In presence of Arochlor-induced rat liver S9 fractions, lilial was almost completely metabolised to lysmerylic acid (RT 18.43 min) and p-tert-butylbenzoic acid (RT 16.34 min) as major metabolites. Additionally, hyroxy-lysmerylic acid (RT 16.11 min) was detected as minor metabolite (Fig. 2) probably as further hydroxylation and oxidation products of lysmerylic acid^39^. In vitro metabolism of ^14^C-lilial using liver microsomes and hepatocytes of rats, rabbits, mice and humans revealed that lysmerol and lysmerylic acid were the primary metabolites, while secondary metabolites included hydroxy-lysmerylic acid and *p*-*tert*-butylbenzoic acid^3,4^. Similar products were detected in urine of 40 human volunteers^39^. However, geometric mean concentrations of 10.21 µg/l for *p*-*tert*-butylbenzoic acid, 1.528 µg/l for lysmeral and below the quantification limit for lysmerylic acid (0.2 µg/l) and hydroxy-lysmerylic acid (0.4 µg/l) were found in the urine of children and adolescents in Germany^40^. *P*-*tert*-butylbenzoic acid has adverse effects on male reproduction in rats^41^. Repeated treatment over 2 weeks with *p*-*tert*-butylbenzoic acid decreased sperm counts, increased abnormal sperm count, and reduced motility^42^. The hydroxy-lysmerylic acid is expected to be not toxic, due to potential further metabolization to, e.g., glucuronic acid conjugates.

### Cytotoxicity

To confirm the lilial concentration range for further assays, we performed the cytotoxic assay on HeLa9903 and CHO-K1 cells. Furthermore, the impact of the S9 fraction which contains a variety of enzymes such as P450 enzymes, carboxylesterases, methyltransferases, and acetyltransferases was also analyzed. These enzymes catalyse the reactions in both phase I and phase II biotransformations in humans and animals. The relative viability of CHO-K1 cells incubated with lilial in the presence or absence of the S9 mixture was determined by resazurin assay after 1 day.

**Figure 3:**
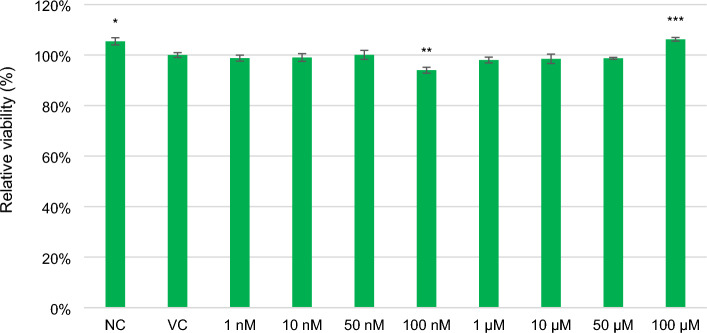
Relative viability of the **HeLa9903** cells (resazurin assay) after 1 day of incubation with lilial at eight concentrations. The final concentration of DMSO was 0.1%, this vehicle control (VC) was taken as 100% viability. Error bars represents for the standard error deviation of six replicates (wells). Statistically evaluated using *t*-test (* indicates statistically significant difference from VC at *p* < 0.05, **p < 0.005, ***p < 0.0005).

Although lilial significantly reduced the relative viability of **HeLa9903** cells at 100 nM (Fig. 3), it was not cytotoxic, as the relative viability was higher than 80% at all concentrations (even the highest concentration up to 100 µM) and no dose–response dependence was observed. According to the ISO 10993-5 standard, a substance is considered cytotoxic if the viability of the cells is less than 70%^43^. The highest concentration chosen roughly corresponds to the maximum allowed concentration in cosmetic preparations of 65 µM.

**Figure 4:**
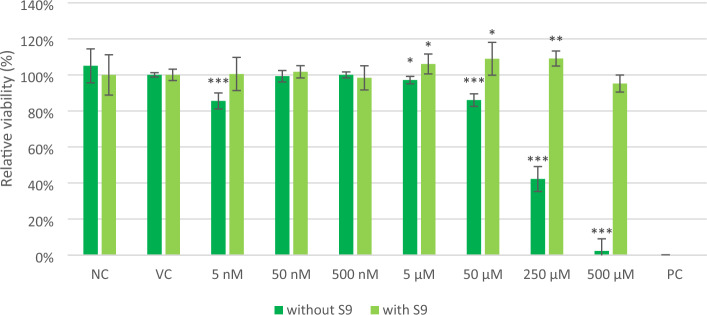
Relative viability of the **CHO-K1** cells (resazurin assay) after 1 day of incubation with lilial at seven concentrations without or with metabolic activation using the S9 mix. The final concentration of DMSO was 0.1%, this vehicle control (VC) was taken as 100% viability. 0.25% Tween-20 served as a positive control (PC). Error bars represents for the standard error deviation of six replicates (wells). Statistically evaluated using *t*-test (*indicates statistically significant difference from corresponding VC at *p* < 0.05, **p < 0.005, ***p < 0.0005).

The relative viability of the **CHO-K1** cells after 1 day of incubation with or without metabolic activation using the S9 mix shows that the non-metabolized lilial significantly decreased the relative viability of CHO-K1 cells approximately by 55 and 95% at 250 and 500 µM, respectively (Fig. 4). However, no cytotoxicity of lilial was observed after metabolization by the S9 mix (even at the highest concentration of 500 µM). The results of our study demonstrate that the cytotoxicity of lilial is reduced after incubation with the metabolizing S9 mix.

The previous study reported a significant decrease (65%) in HaCaT cells viability at a 10 µM concentration of lilial and a 23% decrease in HepG2 and Caco-2 cells viability at a 100 µM concentration of lilial. However, no negative effect was observed on NIH3T3, MCF7 and Hek293 cells^2^.

### Estrogenic and androgenic activity

**Figure 5:**
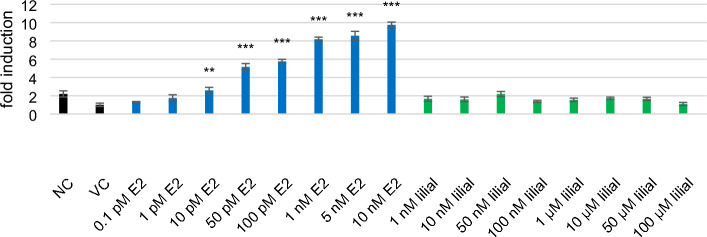
Intensity of response expressed as fold induction (FI) of HeLa9903 cells after 22 h incubation with E2 and lilial. (NC-MEM with 10% DCC-FBS, VC—0.1% DMSO in MEM with 10% DCC-FBS). Error bars indicate the standard error of the mean. Statistically evaluated using ANOVA (R program). *** indicates statistically significant difference from VC at *p* < 0.001, ** difference from VC at 0.001 < *p* < 0.01.

**Figure 6:**
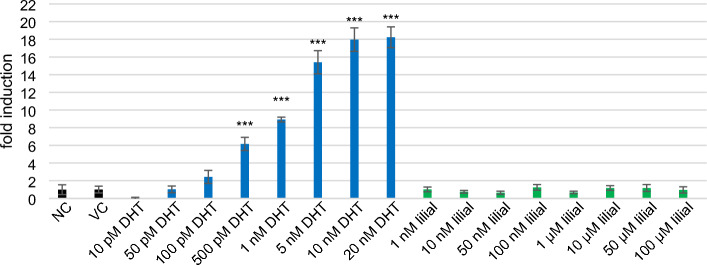
Intensity of response expressed as fold induction (FI) of MDA-kb2 cells after 22 h incubation with DHT and lilial. (NC-RPMI with 10% DCC-FBS, VC—0.1% DMSO in RPMI with 10% DCC-FBS). Error bars indicate the standard error of the mean. Statistically evaluated using ANOVA (R program). *** indicates statistically significant difference from VC at *p* < 0.001.

The endocrine system is a complex network of glands and tissues that produce and secrete hormones to control and coordinate vital body functions such as metabolism, reproduction, growth, stress, sleep, and mood^44^. Endocrine disruptors are compounds that can mimic or interfere with body hormones, resulting in alteration of the hormonal and homeostatic system^45^. To examine the endocrine disruption ability of lilial, we investigated its ability to bind to estrogen and androgen receptors of human cells and act as an agonist of these receptors. The binding ability of lilial to the receptor was compared with E2 and DHT. E2 and DHT significantly increased fold induction (FI) from a concentration of 10 pM and 500 pM, respectively (Figs. 5 and 6). No significant estrogen receptor activation was observed with Lilial in the HeLa9903 cells for the concentrations tested (up to 100 µM). Similarly, no activity was detected in in the MDA-kb2 assay for any of the lilial concentrations (1 nm–100 µM). Therefore, it can be concluded that lilial also does not have androgenic activity. No increase in fold induction was observed after metabolic activation of lilial (Supplementary Figs. 1 and 2). According to Charles and Darbre (2009), lilial possessed very weak estrogenic activity in MCF7 human breast cancer cells in vitro through increased expression of the transfected reporter and endogenous pS2 gene as mentioned in the introduction^8^. According to our results, we were unable to confirm the former suspicion of the European Chemicals Agency (ECHA) that lililal is an endocrine disruptor^1^.

### CHO/HPRT mutation assay and γH2AX biomarker-based genotoxicity assay

The CHO/HPRT mutation assay is a widely used test to detect point and small intragenic mutations in mammalian cells. Its principle is analogous to the bacterial Ames test. Mutations in the *hprt* gene encoding the salvage pathway enzyme hypoxanthine–guanine phosphoribosyl transferase (HPRT) are studied. The toxic purine analogue, 6-thioguanine (6-TG), is used to select mutants of the *hprt* gene^46^. Chinese hamster ovary cells CHO-K1 are used due to their stable karyotype and high cloning efficiency^31^.

The results of the CHO/HPRT mutation assay without and with metabolic activation are listed in Tables 3 and 4, respectively. The substance tested, –lilial, at the concentration tested (500 µM) without metabolic activation was cytotoxic—plating efficiencies (PE) was equal to 0%. This is in agreement with our results with resazurin. Other PE were in a tolerable range (above 50%) including 500 µM lilial after metabolic activation. The VC mutation frequency (MF) was in a tolerable range of 0–20 mutants per 10^6^ cells. Another necessary criterion was also met—PC (240 µg/ml EMS) without metabolic activation induced statistically significant response, i.e. number of mutant colonies (NMC) of PC was significantly higher than of NC (Two Sample *t*-test). Similarly, PC (2 µg/ml 3MC) with metabolic activation induced a statistically significant response, i.e*.* number of mutant colonies (NMC) of PC was significantly higher than that of VC (Two Sample *t*-test).

**Table 3:** Results of CHO-K1/HPRT mutation assay **without activation** expressed as average values of three replicates (plates) ± sample standard deviations.

Sample	NMC	PE (%)	MF (/10^6^)
NC	0 ± 0	95 ± 11	0 ± 0
VC	1 ± 1	103 ± 3	3 ± 3
PC: EMS	10 ± 3	108 ± 13	48 ± 12***
Lilial 50 µM	2 ± 1	117 ± 1	9 ± 4

*VC* vehicle control, *NC* negative control, *PC* positive control, *NMC* number of mutant colonies, *PE* plating efficiency, *MF* mutation frequency, *EMS* ethylmethanesulfonate. Statistical significance was verified by ANOVA followed by Tukey test (*p ˂ 0.05, **p ˂ 0.01, ***p˂ 0.001).

**Table 4:** Results of CHO-K1/HPRT mutation assay **with activation** expressed as average values of three replicates (plates) ± sample standard deviations.

sample	Without S9			With S9		
	NMC	PE (%)	MF (/10^6^)	NMC	PE (%)	MF (/106)
NC	2 ± 1	66 ± 8	**18 ± 9**	**–**	–	–
VC	0 ± 0	69 ± 6	**0 ± 0**	2 ± 2	59 ± 2	**17 ± 17**
PC: 3MC	3 ± 4	66 ± 3	**25 ± 31**	17 ± 2	55 ± 4	152 ± 14***
lilial 500 µM	0	0	**NA**	1 ± 1	56 ± 3	**6 ± 5**

*VC* vehicle control, *NC* negative control, *PC* positive control, *NMC* number of mutant colonies, *PE* plating efficiency, *MF* mutation frequency, *3MC* 3-methylcholanthren. Statistical significance was verified by ANOVA followed by Tukey test (*p ˂ 0.05, **p ˂ 0.01, ***p˂ 0.001).

Significant values are in [bold]

The MF of lilial (50 µM) without metabolic activation and of lilial (500 µM) after metabolic activation was not significantly different from VC (ANOVA followed by Tukey test). Therefore, lilial was not mutagenic to CHO-K1 cells under the conditions used.

For γH2AX biomarker-based genotoxicity assay, we have chosen CHO-K1 cells for comparison with the CHO/HPRT assay and we have chosen also HeLa cells, because they have been used by the manufacturer of the commercial kit used and the HCS γH2AX testing method was also described on them ^47^F. Microscopic images of immuno-stained CHO-K1 and HeLa cells after 4 h and 24 h incubation with lilial with or without S9 mix were analysed as previously described^34^. The mean fluorescence values, MFV (γH2AX signal from Cy3 channel) were extracted using the segmented nuclei from the DAPI channel and expressed as area-scaled relative fold change in fluorescence over negative control, along with changes in cytotoxicity (% control), expressed as relative area (RA). Scaling was adopted from Smart et al.^48^, i.e. a fold change above 1.5 indicated genotoxicity. The results are summarised in Tables 5 and 6. In HeLa, a fold change above 1.5 was observed after 4 h incubation with lilial both with and without the S9 mix. However, the MoA (Mean fluorescence values over Area of the nuclei) values of lilial were not significantly different from those of the negative control. In CHO-K1, after 4 h incubation with lilial with the S9 mix, the fold was 1.50, which is considered equivocal. Nevertheless, in this case, the MoA was significantly higher than the negative control. Lilial without the S9 mix did not increase fold in CHO-K1. Collectively, the ability of lilial to cause double stranded breaks is inconclusive.

**Table 5:** The values from bioimage analysis after 4 h exposition of **HeLa** cell lines to 100 µM lilial with or without the S9 mix compared to unaffected, negative control (NC).

Conditions	Sample	MoA	Fold		RA (%)	
			Mean	IQR	Mean	IQR
4 h	NC	0.05	1.00		100.0	
4 h	Lilial	0.11	**2.15**	1.41	86.1	23.5
4 h	PC	0.13*	**2.63**	0.49	95.8	3.9
4h_S9	NC	0.10	1.00		100.0	
4h_S9	Lilial	0.20	**2.03**	0.26	94.2	4.3
4h_S9	PC	0.29**	**2.94**	0.06	97.8	3.0
24 h	NC	0.02	1.00		100.0	
24 h	Lilial	0.03	1.27	1.34	94.8	23.5
24 h	PC	0.55*	**24.03**	4.33	101.5	3.9

30 μM valinomycin was used as a positive control (PC).

*MoA* mean fluorescence values over area of the nuclei, *IQR* interquartile range, *RA* relative area of nuclei (analogue of relative cell count). Fold values above normative limit of 1.5 are in bold. Statistical significance was verified by Kruskal–Wallis test followed by *post-hoc* Dunn’s test with Bonferroni correction—MoA comparison with NC (*p ˂ 0.05, **p ˂ 0.01, ***p˂ 0.001).

**Table 6:** The values from bioimage analysis after 4 h exposition of **CHO-K1** cell lines to 100 µM lilial with or without the S9 mix compared to unaffected, negative control (NC).

Conditions	Sample	MoA	Fold		RA (%)	
			Mean	IQR	Mean	IQR
4 h	NC	0.80	1.00		100.0	
4 h	Lilial	0.52	0.67	0.05	97.2	3.8
4 h	PC	1.14	1.45	0.17	75.3	4.5
4h_S9	NC	0.73	1.00		100.0	
4h_S9	Lilial	1.10*	1.50	0.13	91.0	4.6
4h_S9	PC	0.99	1.35	0.14	90.8	2.4
24 h	NC	1.18	1.00		100.0	
24 h	Lilial	0.99	0.83	0.07	112.7	6.7
24 h	PC	5.26	**4.43**	2.15	48.5	17.6

30 μM valinomycin was used as a positive control (PC).

*MoA* mean fluorescence values over area of the nuclei, *IQR* interquartile range, *RA* RELATIVE AREA of nuclei (analogue of relative cell count). Fold values above normative limit of 1.5 are in bold. Statistical significance was verified by Kruskal–Wallis test followed by *post-hoc* Dunn’s test with Bonferroni correction—MoA comparison with NC (*p ˂ 0.05, **p ˂ 0.01, ***p˂ 0.001).

The mutagenic activity of lilial has been evaluated using Ames test at a concentration of up to 5000 µg/plate with or without metabolic activation. An increased number of revertant colonies were detected only for the *Salmonella* strain TA1535, but it was not reproducible in a confirmatory plate incorporation test^4^. Overall, the majority of mutagenicity results in bacteria do not provide the possibility of the mutagenic potential of lilial^3^; however, despite the fact that it is a standardized test of first choice, in many cases it may not be sufficient and therefore verification is necessary using tests based on mammalian cells. In a mammalian cell gene mutation assay conducted in Chinese hamster lung and human colonic epithelial cells, lilial was not mutagenic to mammalian cells in vitro^3,5^. Similarly, a negative outcome was observed in the mouse lymphoma assay^4^.

### Nephrotoxicity

As the primary route of entry of lilial is the skin or the nasal mucosa, the probability of ITS toxic effect on the kidneys is very low. Nevertheless, it is a very sensitive test that has a great informative value and can also indicate other mechanisms of action. Therefore, the lily was also subjected to the detection of nephrotoxic potential. Nephrotoxicity was evaluated as an ability of lilial and its metabolites to alter the expression profile of biomarkers that indicate kidney damage. Biomarkers were chosen based on previous works^49–51^. All data were normalized to the expression of both the *GAPDH* and *ACTB* gene. The experiment was carried out in three biological repetitions, which were after then divided into the three technical repetitions. Relative fold gene expression was calculated to the expression of each normalization gene independently. After that, the results are presented as an average of both normalization genes. Based on our results, lilial is not toxic up to the 100 µM concentration (Table 7). Lilial significantly decreased the expression of the metallopeptidase inhibitor 1 (*TIMP1*) and cystatin C (*CST3*) genes. The decrease in *CST3* gene expression was also statistically significant in the metabolically activated sample. Acute renal failure is defined by a rapid decrease in glomerular filtration rate, which is associated with an increase in cystatin C^52^. Therefore, the decrease in *CST3* gene expression could result in a decrease in cystatin C, which is in opposite to kidney damage. On the other hand, lower levels of cystatin C can be associated with an increased risk of Alzheimer’s and aneurysms^53^. Similarly, decreased expression of TIMP-1 leads to withdrawal of neuroprotection and increased neuronal dysfunction^54^.

**Table 7:** Relative fold gene expression of hTERT-immortalized epithelial proximal tubular cells (RPTEC/TERT1) treated with 100 µM lilial or its metabolically activated form (lilial with S9).

Gene	Lilial		Lilial with S9		Vehicle control		Tetracycline	
	Fold	SD	Fold	SD	Fold	SD	Fold	SD
*HMOX*	1.19	0.50	0.94	0.05	1.02	0.02	4.93**	0.38
*TIMP1*	0.33*	0.11	0.86	0.01	1.16	0.12	1.31	0.04
*IPO8*	0.95	0.41	0.96	0.02	1.02	0.02	1.41**	0.02
*IL6*	1.09	0.43	1.21*	0.03	1.03	0.03	1.70	1.30
*CST3*	0.33*	0.10	0.78*	0.00	1.06	0.06	1.64*	0.10
*IL8*	0.59	0.19	0.94	0.02	1.14	0.11	1.19	0.02
*CLU*	1.10	0.37	1.05	0.09	1.10	0.09	4.09*	0.39

DMSO was used as a vehicle control in the same concentration as in samples.

Tetracycline (100 µM) was used as positive control known for its nephrotoxicity.

Significant values are in [bold].

Metabolic activation of the lilial resulted in a statistically significant increase in *IL-6* gene expression. The *IL-6* gene encodes a cytokine that functions in inflammation and the maturation of B cells. In addition, IL-6 is an endogenous pyrogen capable of inducing fever in people with autoimmune diseases or infections. The functioning of this gene is involved in a wide variety of inflammation-associated disease states, including susceptibility to diabetes mellitus and systemic juvenile rheumatoid arthritis. IL-6 is commonly increased in patients with chronic kidney disease, which is largely caused by the increase in generation resulting from oxidative stress, chronic inflammation, and fluid overload^55^. In conclusion, neither lilial, nor its metabolites, demonstrated nephrotoxic activity up to the 100 µM concentration using epithelial proximal tubular cells.

### Signal pathways activation

The main cellular pathways that maintain redox balance and control the response to oxidative stress and inflammation are associated with the transcription factors NRF2 and NF-κB^56^. Translocation of NRF2 to the nucleus is prevented by binding to Keap1, which can release NRF2 in the presence of oxidative stress or xenobiotics within the cell^57^. After translocation of NRF2 to the nucleus and binding to other transcription factors, the synthesis of phase II antioxidant and detoxification enzymes occurs^58^. NF-κB can regulate the cellular stress response by releasing inflammatory cytokines and other molecules^59^. In addition, it has the ability to regulate apoptosis and can function as both a pro-apoptotic factor and an anti-apoptotic factor^60^.

**Figure 7:**
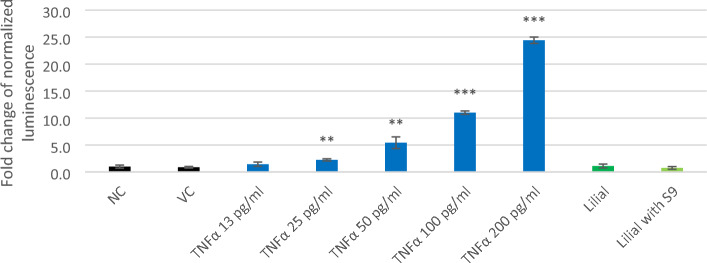
Induction of NF-kB pathway expressed as fold induction of transfected HEK293 cells treated with TNFα (positive control), lilial, and lilial activated with the S9 (both 100 µM). (NC—untreated cells, VC—DMSO). Error bars indicate the standard error of the mean. Statistically evaluated using *t*-test (*indicates statistically significant difference from NC at *p* < 0.05, **p < 0.005, ***p < 0.0005).

**Figure 8:**
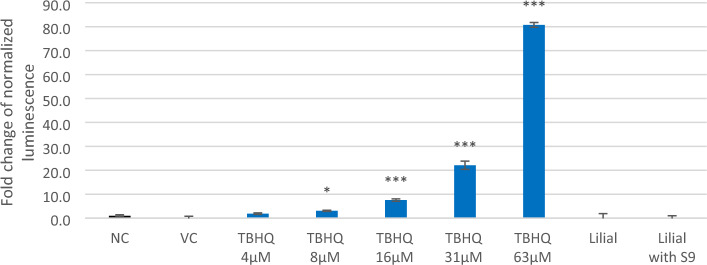
Induction of NRF2-ARE pathway expressed as fold induction of transfected HEK293 cells treated with tert-butylhydroquinone (TBHQ, positive control), lilial, and lilial activated with the S9 (both 100 µM). (NC—untreated cells, VC—DMSO). Error bars indicate the standard error of the mean. Statistically evaluated using *t*-test (*indicates statistically significant difference from NC at *p* < 0.05, **p < 0.005, ***p < 0.0005).

In the study by Ade et al.^15^, a significant increase in the level of NRF2 protein in THP-1 cells treated with lilial (650 µM) for 5 h suggested that the NRF2 pathways are activated by lilial. Lilial was negative in the KeratinoSens assay (Natsch et al.^18^). However, based on the review of the literature, lilial after metabolic activation has not been studied in this manner, i.e. there are no publications on the lilial metabolites-activated NRF2 and NF-κB pathways. Also in our study, neither the NF-kB (Fig. 7) nor NRF2 (Fig. 8) signalling pathway was activated by lilial or its metabolites.

## Conclusion

Despite the recent ban on the use of lilial in the EU, there is no consensus in the literature regarding its toxicological properties. A number of authors contradict each other. Based on in vitro data with the Caco-2 model, lilial may be able to penetrate the intestinal membrane. However, major uptake route of lilial is via the skin, thus oral uptake is of no relevance. Lilial did not show cytotoxicity, genotoxicity, nephrotoxicity or the ability to disrupt the endocrine system in vitro in relevant concentrations (up to 100 µM). For a possible more detailed assurance, it would be advisable to verify that lilial is not an antagonist of the mentioned receptors and that it does not interact with the thyroid system. Considering all our negative results in toxicological tests in vitro, the likely mechanism of action will be that the metabolite p-tert-butylbenzoic acid is produced in the organism, which further conjugates with coenzyme A and interferes with lipid metabolism as recently demonstrated by^42^.

### Supplementary Information

Supplementary Figures.

## Data Availability

The datasets used and/or analysed during the current study available from the corresponding author on reasonable request.
