# Supplementary material for: Toxicological investigation of lilial
Source: Sci Rep. 2023 Oct 28;13:18536. doi: 10.1038/s41598-023-45598-y (PMC10613275; doi:10.1038/s41598-023-45598-y)
Supplement: Supplementary file 1 — Supplementary Figures. [file 41598_2023_45598_MOESM1_ESM.docx]

# Toxicological investigation of lilial

*Eva Jablonská^1^, Zdeněk Míchal^1^, Bára Křížkovská^1^, Ondřej Strnad^1^, Van Nguyen Tran^1^, Tereza Žalmanová^2^, Jaroslav Petr^2^, Jan Lipov^1^, Jitka Viktorová^1*^*

1 Department of Biochemistry and Microbiology, University of Chemistry and Technology, Prague, Technická 5, 166 28 Prague 6, Czech Republic

2 Department of Biology of Reproduction, Institute of Animal Science, Prague 10-Uhrineves, Czech Republic

* Correspondence: prokesoj@vscht.cz

**Supplementary Figure 1:** Intensity of response expressed as fold induction (FI) of HeLa9903 cells after 22h incubation with E2, lilial and activated lilial. (NC – MEM with 10% DCC-FBS, VC – 0.1% DMSO in MEM with 10% DCC-FBS). Error bars indicate the standard error of the mean. Statistically evaluated using ANOVA (R program). *** indicates statistically significant difference from VC at p < 0.001, ** difference from VC at p < 0.01.

**Supplementary Figure 2:** Intensity of response expressed as fold induction (FI) of MDA-kb2 after 22h incubation with DHT, lilial and activated lilial. (NC – MEM with 10% DCC-FBS, VC – 0.1% DMSO in MEM with 10% DCC-FBS). Error bars indicate the standard error of the mean. Statistically evaluated using ANOVA (R program). *** indicates statistically significant difference from VC at p < 0.001, ** difference from VC at p < 0.01.
